# Short-term high-fat diet consumption impairs synaptic plasticity in the aged hippocampus via IL-1 signaling

**DOI:** 10.1038/s41538-023-00211-4

**Published:** 2023-07-17

**Authors:** Brigitte M. González Olmo, Menaz N. Bettes, James W. DeMarsh, Fangli Zhao, Candice Askwith, Ruth M. Barrientos

**Affiliations:** 1 Department of Biomedical Education & Anatomy, Ohio State University, Columbus, OH USA; 2 Institute for Behavioral Medicine Research, Ohio State University, Columbus, OH USA; 3 Department of Neuroscience, The Ohio State University, Columbus, OH USA; 4 Department of Psychiatry and Behavioral Health, Ohio State University, Columbus, OH USA; 5 Chronic Brain Injury Program, The Ohio State University, Columbus, OH USA

**Keywords:** Neuroimmunology, Membrane potential, Risk factors

## Abstract

More Americans are consuming diets higher in saturated fats and refined sugars than ever before. These trends could have serious consequences for the older population because high-fat diet (HFD) consumption, known to induce neuroinflammation, has been shown to accelerate and aggravate memory declines. We have previously demonstrated that short-term HFD consumption, which does not evoke obesity-related comorbidities, produced profound impairments to hippocampal-dependent memory in aged rats. These impairments were precipitated by increases in proinflammatory cytokines, primarily interleukin-1 beta (IL-1β). Here, we explored the extent to which short-term HFD consumption disrupts hippocampal synaptic plasticity, as measured by long-term potentiation (LTP), in young adult and aged rats. We demonstrated that (1) HFD disrupted late-phase LTP in the hippocampus of aged, but not young adult rats, (2) HFD did not disrupt early-phase LTP, and (3) blockade of the IL-1 receptor rescued L-LTP in aged HFD-fed rats. These findings suggest that hippocampal memory impairments in aged rats following HFD consumption occur through the deterioration of synaptic plasticity and that IL-1β is a critical driver of that deterioration.

## Introduction

Consumption rates of high-fat, high-sugar, and high-refined carbohydrate (semi- and ultra-processed) foods, collectively known as the Western diet, remain quite high and correlate well with high rates of overweight and obesity^1,2^. The Western diet has been tightly correlated with an increase in chronic disease prevalence (e.g., cardiovascular disease, type 2 diabetes, infertility)^3–5^, and has been shown to negatively impact human digestive physiology and have pathogenic-like effects on the immune system^6,7^. Furthermore, chronic high-fat diet (HFD) consumption is associated with exaggerated inflammation and linked with impairments in memory and increased risk for neurodegenerative diseases^8–11^. Owing to the fact that these obesity-related comorbidities also interfere with memory-forming processes, determining specific mechanisms linking unhealthy diet to memory deficits has been difficult and confounded. More recently, compelling research in humans indicates cognitive impairments can occur even after acute (1–7 days) consumption of HFD^12–15^, suggesting that dietary components play an important role in cognitive functions as they are capable of rapidly interacting with the central nervous system, even in the absence of obesity.

Normal aging is associated with gradual memory declines but when combined with physical insults (e.g., surgery, infection, or injury) that evoke a neuroinflammatory response, memory declines become accelerated and aggravated^16–19^, increasing vulnerability to neurodegenerative diseases such as Alzheimer’s disease^20^. This exacerbated neuroinflammatory response may be due to the observation that healthy aged rodents (20–24 months old) exhibit a steady-state sensitized microglial phenotype that is characterized by increased expression of immunological and morphological markers, and a lowered reactivity threshold compared to microglia in healthy young adult rodents (3–6 months old)^21,22^. Thus, in response to an inflammatory insult, these sensitized microglia become hyper-reactive and produce persistent and exaggerated levels of proinflammatory cytokines^23–25^. Healthy young adult rodents, whose microglia are not sensitized at baseline, do not exhibit similarly exaggerated inflammatory responses^21,23^. Furthermore, the aged brain exhibits increased astrocytic activity and hypertrophy^26,27^, which is associated with progressive reactivity of microglia in aging^28^.

We have previously demonstrated that a short-term (three days) regimen of HFD consumption is sufficient to produce an exaggerated increase in proinflammatory cytokines, particularly interleukin-1 beta (IL-1β), in the hippocampus and amygdala of aged, but not young adult rats^18^. This HFD regimen also led to significant impairments in hippocampal- and amygdalar-dependent long-term memory (as measured by contextual freezing 4 days post conditioning) but not short-term memory (measured 1–2 h post conditioning), in aged rats, and did not produce any memory impairments in young adults^18^. In young rats, an additional mild endotoxin challenge was needed to provoke an exaggerated neuroinflammatory response, and impair long-term memory^29^. Importantly, these neuroinflammatory and cognitive effects induced in aged rats after just 3 days on HFD were observed in the absence of diet-induced obesity-related comorbidities such as glucose or insulin disturbances^18^. These findings prompted us to examine the extent to which long-term memory formation mechanisms may be impaired in aged rats following short-term HFD consumption.

Research suggests that abrupt aging-related deficits in long-term episodic memory are often a result of alterations in synaptic efficacy and plasticity^30^. In addition, exaggerated neuroinflammation is known to impair synaptic plasticity as measured by long-term potentiation (LTP). LTP is a form of synaptic plasticity characterized by increases in excitatory postsynaptic potentials following a robust stimulation of presynaptic neurons, that occurs in multiple brain regions and has long been considered to be the cellular mechanism for learning and memory^31^. In the hippocampus, LTP has been extensively studied in all afferent pathways^32^. Also, different phases of LTP have been described. For example, early-phase LTP (e.g., E-LTP) is induced with a single short, high-frequency stimulation, involves the modification of existing proteins, and correlates to short-term memory. In contrast, late-phase LTP (e.g., L-LTP) is induced with repeated, short stimulation trains in the theta frequency range with short interstimulus intervals (termed theta-burst stimulation), requires de novo protein synthesis to produce sustained firing, and correlates to long-term memory^33^. Chronically elevated IL-1β impairs hippocampal LTP, and there is evidence that this occurs through multiple mechanisms, including inhibition of glutamate release^34^, dampening of brain-derived neurotrophic factor (BDNF)^35^, inhibition of Akt/mTOR signaling^36^, downregulation of GluA1, an AMPA receptor subunit, and LIMK1, a kinase that promotes actin polymerization^36^, and upregulation of reactive oxygen species^37^. Thus, our previous work led us to hypothesize that the combination of aging and short-term HFD consumption would evoke a profound impairment in L-LTP in the hippocampus. We further hypothesized that this impairment would be mediated by elevated levels of IL-1β.

To achieve the goals of this study, we performed three different experiments with separate cohorts (specific details can be found in the Methods section below). Briefly, in experiment 1, we determined the extent to which short-term HFD consumption would disrupt long-lasting forms of synaptic plasticity (L-LTP) in the young adult and aged hippocampus. In experiment 2, we determined the extent to which short-term forms of synaptic plasticity (E-LTP) would be impaired in aged rats following HFD. Lastly, in experiment 3, we determined the extent to which central IL-1β mediates HFD-induced L-LTP disruptions in aged rats. We hypothesized that long-lasting forms of synaptic plasticity would be significantly impaired in aged HFD-fed rats compared to all other groups. We further hypothesized that impairments in short-term forms of synaptic plasticity (E-LTP) would not be observed, given the lack of a short-term memory deficit in these rats in previous studies. Finally, we hypothesized that the synaptic disruptions observed in L-LTP in aged HFD-fed rats would be rescued by blockade of the IL-1 receptor with the interleukin-1 receptor antagonist (IL-1RA).

## Results

As mentioned below in “Methods”, only one animal was processed per day due to limited equipment availability. Thus, the second animal in the cage remained on their assigned diet an extra day. To confirm that this additional day on the HFD had not skewed the data, a repeated measures ANOVA was run between all “first” and “second” rats for each measure. Results indicated no significant differences (*P* > 0.05) for all experiments.

### Experiment 1: HFD disrupts late-phase LTP in the hippocampus of aged rats

**Fig. 1:**
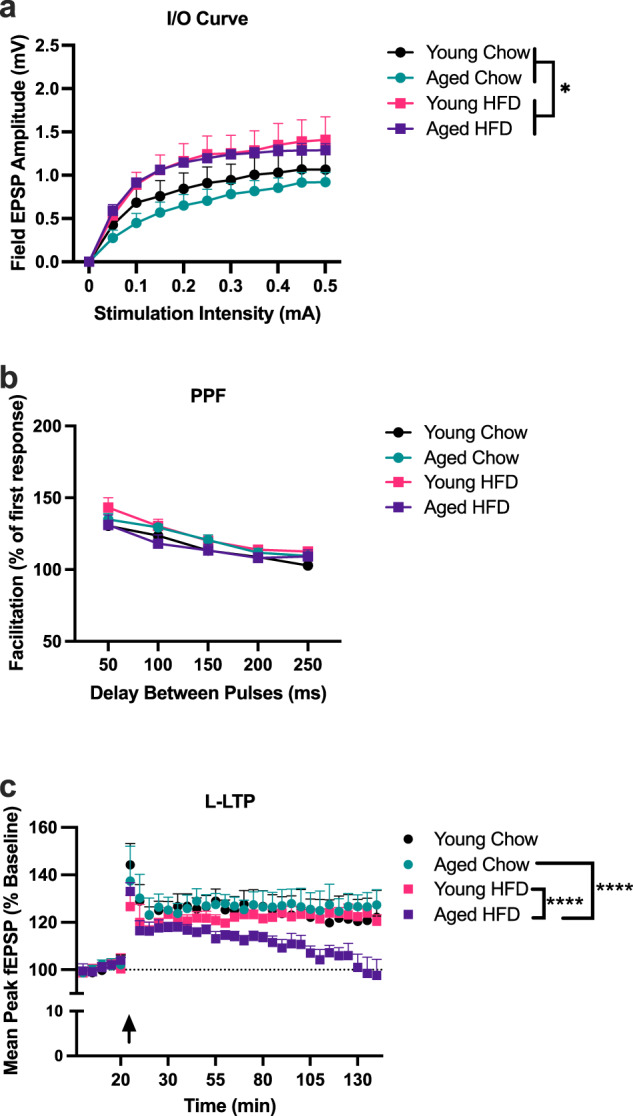
Theta-burst L-LTP is impaired by the combination of aging and HFD. Slices from young chow (9 slices, 6 rats), young HFD (6 slices, 5 rats), aged chow (10 slices, 6 rats), and aged HFD (6 slices, 4 rats) were analyzed using two-way repeated measures ANOVAs. **a** Input/Output curves indicated significant elevations in slices from HFD-fed rats compared to chow-fed rats, irrespective of age (*P < 0.05). **b** Paired-pulse facilitation was not significantly altered by age or diet at any of the inter-pulse intervals. **c** L-LTP was significantly degraded in aged HFD-fed rats compared to aged chow-fed and young HFD-fed rats (****P < 0.0001). No other groups differed from each other. Error bars represent SEM.

We began by examining basal synaptic transmission at the Schaffer collateral–CA1 synapses in hippocampal slices. We generated input–output curves for each slice to indicate possible differences in response to stimuli of a given of intensity (and also set the suitable stimulation intensity to conduct LTP). A 2-way repeated measures ANOVA indicated there was a main effect of diet (*F*_(1,26)_ = 5.71, *P* < 0.05) but no main effect of age (*F*_(1,26)_ = 0.496, *P* > 0.05). There was, as expected a main effect of stimulation intensity with greater EPSP amplitude with increasing stimulation intensities (*F*_(9234)_ = 81.67, *P* < 0.0001); however, there was no interaction effect between condition and stimulation intensity (*F*_(9207)_ = 0.48, *P* > 0.05; Fig. 1a). We next examined paired-pulse facilitation (PPF), a measure of presynaptic short-term plasticity, in each slice^38^. PPF is believed to reflect the residual Ca^2+^ in the presynaptic nerve terminal from the first stimulus adding up to the influx of Ca^2+^ evoked by the second stimulus, thus resulting in an increase of neurotransmitter release^39^. Results from a two-way repeated measures ANOVA demonstrated that PPF was not significantly different across age (*F*_(1,33)_ = 1.19, *P* > 0.05), diet (*F*_(1,33)_ = 1.18, *P* > 0.05), nor was there an age x diet interaction (*F*_(1,33)_ = 1.19, *P* > 0.05; Fig. 1b). We then proceeded to investigate L-LTP for each slice using the theta-burst (TBS) protocol, recognized as a more robust LTP induction paradigm, designed to mimic the burst firing of CA1 pyramidal cells at theta frequency recorded in vivo from awake animals during spatial exploration^40–42^. This protocol is considered to be more naturalistic and has proven to be a sensitive indicator of alterations in mnemonic processes associated with aging^43,44^. Results from a two-way repeated measures ANOVA indicated no main effect of age (*F*_(1,18)_ = 3.06, *P* > 0.05), a main effect of diet (*F*_(1,18)_ = 4.56, *P* < 0.05), and a significant diet × age × time interaction (*F*_(25,450)_ = 7.26, *P* < 0.0001); Fig. 1c). Scheffe’s post hoc analyses revealed that aged HFD-fed rats had significantly lower EPSPs over time compared to diet-matched young controls (*P* < 0.0001) and age-matched chow controls (*P* < 0.0001). There were no other group differences.

### Experiment 2: HFD does not disrupt early-phase LTP in the hippocampus of aged rats

**Fig. 2:**
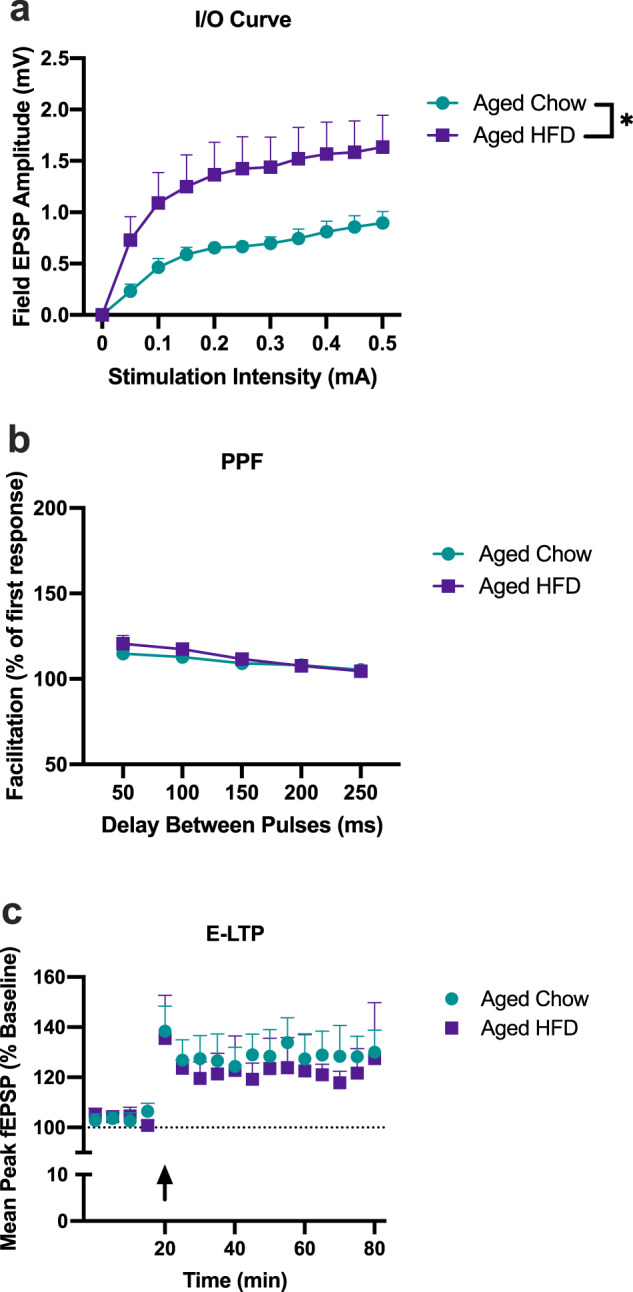
E-LTP is not impaired by the combination of aging and HFD. Slices from aged chow (8 slices, 6 rats), and aged HFD (8 slices, 6 rats) were analyzed using two-way repeated measures ANOVAs. **a** Input/Output curves indicated significant elevations in slices from HFD-fed rats compared to chow-fed rats (*P < 0.05). **b** Paired-pulse facilitation was not significantly altered by diet at any inter-pulse interval. **c** Early-phase LTP was not significantly altered by diet. Error bars represent SEM.

To examine the extent to which diet alters short-term plasticity in these rats, we used a single high-frequency stimulus train: 1 s, at 100 Hz protocol, known to induce E-LTP. Given our previous findings demonstrating that young adult rats do not exhibit elevated neuroinflammation nor any memory impairments in response to HFD^18^, and the results of Experiment 1 of the current study indicating that L-LTP EPSPs were not significantly altered with HFD in young adult rats, only aged rats were examined going forward. All data for this experiment were analyzed using two-way repeated measures ANOVAs. We generated input–output curves for each slice to indicate possible differences in the response to stimuli of a given of intensity. There was a main effect of diet (*F*_(1,14)_ = 5.062, *P* < 0.05), with slices from HFD-fed rats exhibiting greater amplitudes than those from chow-fed rats (Scheffe’s post hoc: *P* < 0.0001). There was, as expected, a main effect of stimulation intensity with greater EPSP amplitude with increasing stimulation intensities (*F*_(9126)_ = 39.17, *P* < 0.0001); however, there was no interaction effect between condition and stimulation intensity (*F*_(9126)_ = 0.64, *P* > 0.05; Fig. 2a). PPF results demonstrated that there were no significant differences between diet (*F*_(1,14)_ = 0.24, *P* > 0.05; Fig. 2b), nor was there a diet by pulse interval interaction (*F*_(4,56)_ = 0.47, *P* > 0.05; Fig. 2b). E-LTP results indicated no main effect of diet (*F*_(1,14)_ = 0.38, *P* > 0.05) and no diet × time interaction (*F*(_11,125_) = 0.37, *P* > 0.05; Fig. 2c).

### Experiment 3: Blockade of IL-1 receptor with IL-1RA rescues L-LTP in aged HFD-fed rats

**Fig. 3:**
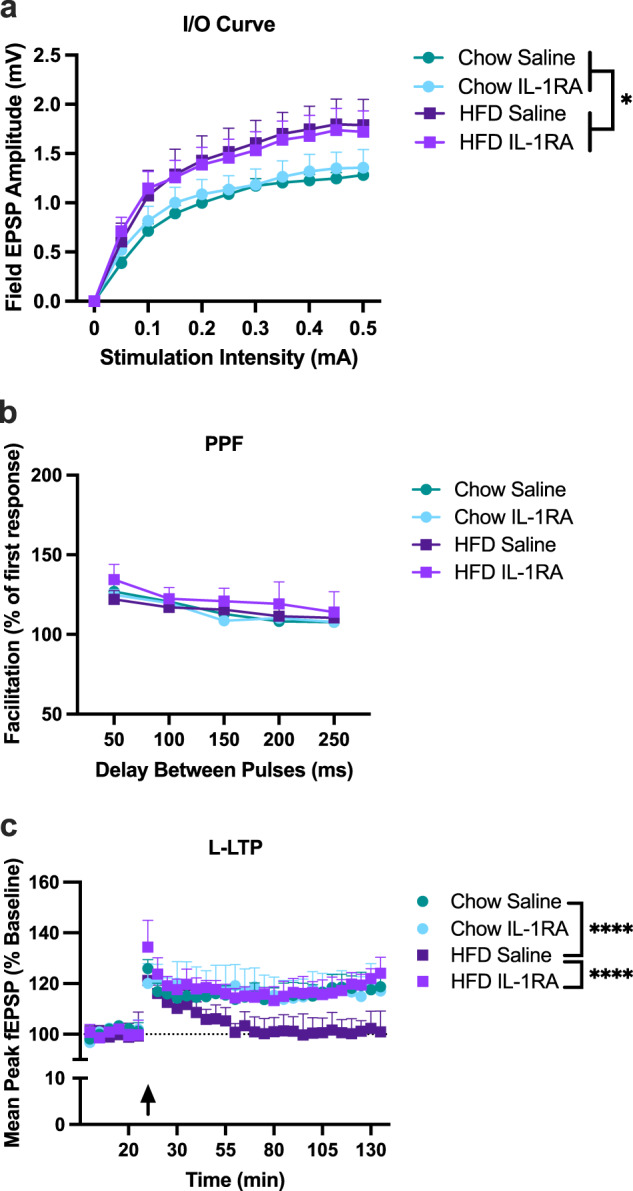
HFD-induced deficits in theta-burst L-LTP is rescued by blocking IL-1 signaling with IL-1RA. Slices examined came from aged rats from the following groups: chow saline (9 slices, 5 rats), chow IL-1RA (7 slices, 6 rats), HFD saline (7 slices, 5 rats), and HFD IL-1RA (8 slices, 6 rats) and were analyzed using two-way repeated measures ANOVAs. **a** Input/Output curves indicated significant elevations in slices from HFD-fed rats, compared to chow-fed rats, irrespective of drug (*P < 0.05). **b** Paired-pulse facilitation was not significantly altered by diet or drug at any inter-pulse interval. **c** L-LTP was significantly degraded in saline-treated HFD-fed rats compared to chow-fed controls (****P < 0.0001) and IL-1RA treated HFD-fed rats (****P < 0.0001). IL-1RA-treated HFD-fed rats did not differ from either chow-fed control rats. Error bars represent SEM.

As mentioned earlier, our previous work has demonstrated that HFD consumption elicits a significant increase in the proinflammatory cytokine IL-1β within the aged hippocampus, and that blocking that increase with the IL-1 receptor antagonist (IL-1RA) prevents HFD-induced memory deficits in aged rats^18^. Elevated levels of IL-1β in the hippocampus have been repeatedly demonstrated to impair synaptic plasticity^35,44–48^, but whether short-term HFD-induced elevated IL-1β levels caused synaptic plasticity impairments in aged rats has never been determined. Thus, to determine the extent to which HFD-induced L-LTP disruptions in aged rats would be ameliorated by blocking IL-1β signaling, here we injected IL-1RA centrally, prior to HFD consumption. All data for this experiment were analyzed using two-way repeated measures ANOVAs. Input–output curves indicated a main effect of diet (*F*_(1,24)_ = 6.12, *P* < 0.05), with HFD again exhibiting significantly elevated amplitudes compared to chow-fed controls (Scheffe’s post hoc: *P* < 0.0005). There was no main effect of drug (*F*_(1,24)_ = 0.31, *P* > 0.05). There was, as expected a main effect of stimulation intensity with greater EPSP amplitude with increasing stimulation intensities (*F*_(9216)_ = 170.86, *P* < 0.0001); however, there was no interaction effect between condition and stimulation intensity (*F*_(9216)_ = 0.25, *P* > 0.05; Fig. 3a). PPF results demonstrated no significant differences across diet (*F*_(1,27)_ = 0.547, *P* > 0.05), drug (*F*_(1,27)_ = 0.304, *P* > 0.05), nor was there an interaction (*F*_(4108)_ = 0.350, *P* > 0.05; Fig. 3b). L-LTP results indicated no main effect of diet (*F*_(1,18)_ = 2.80, *P* > 0.05), no main effect of drug (*F*_(1,18)_ = 3.31, *P* > 0.05), but a significant diet x drug × time interaction (*F*_(24,432)_ = 3.76, *P* < 0.0001); Fig. 3c. Scheffe’s post hoc analyses revealed that HFD-fed saline-treated rats exhibited significantly lower EPSPs over time compared to chow-fed controls (*P* < 0.0001), replicating findings of Experiment 1. However, HFD-fed rats treated with IL-RA exhibited EPSPs that were significantly higher than those of HFD-fed saline-treated controls (*P* < 0.0001) and were indistinguishable from EPSPs of all other groups (*P* > 0.05).

## Discussion

Our results indicate that L-LTP was significantly compromised in the hippocampus of HFD-fed aged rats. Importantly, this long-lasting synaptic plasticity was robustly maintained in HFD-fed young adult rats and chow-fed rats of either age, indicating that the combination of both advanced age and HFD is necessary to result in negative outcomes. In contrast, E-LTP, a cellular correlate of short-term memory^49^ was not altered by HFD. Together, these data are in agreement with our previous findings that long-term memory, but not short-term memory, were impaired in aged rats following 3 days of HFD^18^.

Results further showed that a single central injection of IL-1RA prior to the first HFD meal was sufficient to rescue the HFD-induced L-LTP deficit. It should be noted that the profoundly degraded L-LTP exhibited in slices from saline-treated rats fed HFD in this experiment served to replicate initial findings of Experiment 1, that HFD robustly deteriorates L-LTP in aged rats. It is not surprising that a single injection was sufficient to ameliorate the HFD-induced IL-1β effects, as we have previously shown that IL-1RA injected centrally, via cisterna magna, has a half-life of 4 days^50,51^, a stark contrast with the 1.5-h half-life of the drug when injected peripherally^51,52^. This robust L-LTP rescue effect parallels our previous findings showing that a central injection of IL-1RA ameliorated HFD-induced long-term memory impairments in aged rats^18^. Taken together, these findings strongly support the conclusion that the previously observed IL-1β-mediated hippocampal memory impairments in aged HFD-fed rats occurs through deterioration of synaptic plasticity and that IL-1β is a critical driver of that deterioration.

As mentioned earlier, several mechanisms by which exaggerated IL-1β inhibits LTP have been described. For example, alterations of glutamate receptor subunits and glutamate release^34,36^, blunting of BDNF^35,53–55^, inhibition of Akt/mTOR signaling^36^, and increased reactive oxygen species production^37^ are all direct consequences of elevated IL-1β that contribute to deteriorated synaptic plasticity. Moreover, a more recent study using ex vivo methods has demonstrated that IL-1β suppresses LTP directly at synapses via neuron-specific mechanisms, whereas other proinflammatory cytokines (IL-18) require indirect mechanisms involving microglia and astrocytes^47,56^. Whether the observed IL-1β-mediated hippocampal memory impairments in the aged HFD-fed rats might be occurring through one or more of these mechanisms and whether they are impacting neurons directly or indirectly remains to be examined.

Neuronal input–output function refers to the relationship between the excitatory input to a neuron and the probability it will generate an action potential, and is a measure of basal synaptic transmission^56–59^. Surprisingly, HFD, compared to chow, caused significant increases in basal synaptic transmission, independent of age or drug. The existing literature offers few insights on potential factors that might lead to increases in basal synaptic transmission despite drastically degraded long-term synaptic plasticity. A possible explanation lies with the role of astrocytes as endogenous regulators of basal synaptic transmission^60,61^. One study demonstrated that astrocytes performed local processing of synaptic information at the level of the synapse^60^. Specifically, it was demonstrated that astrocytes in the CA1 region of the hippocampus detected activity and upregulated basal synaptic transmission^60^. Another study confirmed intense local Ca^2+^ activity in the processes of mature astrocytes in the dentate gyrus^62^. It was proposed that most likely, these Ca^2+^ signals were not involved in triggering gene expression in the nucleus but instead exerted a local regulatory influence on synaptic function^62^. Interestingly, a long-term HFD study using young adult mice found increased lipid content and changes in the metabolism of hippocampal astrocytes but not neurons, indicating that astrocytes in the hippocampus are a primary target of fatty acids^63^. Thus, it is possible that HFD might evoke astrocyte-induced increased basal synaptic transmission, as reflected in our data, but degrade long-lasting synaptic plasticity via other mechanisms discussed above. Examining the role of astrocytes in basal transmission and L-LTP following HFD, especially in aged rats is a future direction of this study.

As mentioned in “Methods”, the current work was done exclusively in male rats due to unavailability of aged female rats at the NIA colony at the time these experiments were conducted. Thus, it is important to use caution when interpreting these data, as given the well know sex differences in inflammatory-related diseases, these results could differ in female rats. It has been demonstrated that young adult females exhibit a decreased neuroinflammatory profile and are protected from insults to learning and memory^64^, but aging females may be at greater risk for neuroinflammation and cognitive decline than males, as there is an increase in Alzheimer’s disease prevalence in older women than men^65^. Future experiments will examine synaptic function following HFD in young adult and aged female rats.

Taken together, this work demonstrates that short-term consumption of HFD is sufficient to impair long-lasting hippocampal synaptic plasticity in aged rats and that it does so via elevations in IL-1β. Future studies will aim to investigate the extent to which HFD affects other regions of the brain such as the amygdala, the role of astrocytes on HFD-induced basal transmission, and will explore nutritional (DHA) and behavioral (voluntary exercise) interventions, known to blunt central IL-1β elevations^53,66–69^, to ameliorate synaptic plasticity dysregulation in the aged brain following HFD consumption.

## Methods

### Animals

Subjects were young adult (3-month-old) and aged (24-month-old) male F344×BN F1 rats obtained from the National Institute on Aging Rodent Colony maintained by Charles River. Unfortunately, female rats of this strain and ages, exclusively available through the NIA colony, were not available at the time these studies were conducted. Condition-matched rats were housed 2 per cage (52 L × 20 W × 21H, cm). The animal colony was maintained at 22 °C on a 12-h light/dark cycle (lights on at 07:00 h). Animals were allowed free access to food and water and were given at least 1 week to acclimate to colony conditions before experimentation began. All experiments were conducted in accordance with protocols approved by the Ohio State University Institutional Animal Care and Use Committee. Every effort was made to minimize the number of animals used and their suffering.

### Diet

A total of 55 rats were used in this study. At study onset, young and aged animals were randomly assigned to either continue consuming their regular chow (Inotiv TD.8640; energy density of 3.0 kcal/g; 29% calories from protein, 54% from carbohydrates [no sweetener added], and 17% from fat [0.9% saturated, 1.2% monounsaturated, 2.7% polyunsaturated]), or an adjusted calorie 60% HFD (Inotiv TD.06414, energy density of 5.1 kcal/g; 18.4% calories from protein, 21.3% from carbohydrates [90 g/kg sucrose, 160 g/kg maltodextrin], and 60.3% from fat [37% saturated, 47% monounsaturated, 16% polyunsaturated]). Rats consumed HFD ad libitum for 3 or 4 days. Only one animal per cage could be processed per day due to limited equipment availability and because these animals were pair-housed, the second animal in the cage received an extra day of diet.

### Tissue collection

For all experiments, rats were rapidly decapitated without anesthesia prior to tissue collection. Brains were carefully extracted from the skull and placed in an ice-cold slicing solution (in mM: 250 sucrose, 24 NaHCO_3_, 25 glucose, 2.5 KCl, 1.25 NaH_2_PO_4_, 1.5 MgSO_4_, 2 CaCl_2_; pH adjusted to 7.3–7.4) for ~20 min prior to preparing for slicing.

### Slice preparations

For all experiments, transverse hippocampal slices were collected using a vibratome. The brain was transferred to a cold Petri dish with the same ice-cold slicing solution (described above). To obtain hippocampal sections, the cerebellum and a small part of the prefrontal cortex were cut off. The remaining block was cut along the midline into two equal hemispheres using a scalpel blade. Both hemispheres were mounted to the specimen holder with superglue and a supporting piece of agar behind the brain, away from the side of the vibratome, to provide structural support during slicing. The vibratome was set up by filling the buffer tray with ice-cold slicing solution, ice bath tray with ice to keep the buffer tray cold during slicing, and adjusting the desired thickness to 400 μm and cutting speed to 0.06 mm/s. Using a transfer pipette, slices were transferred to the slice incubation chamber that was placed in a water bath to be maintained at 28 °C and perfused with oxygenated aCSF (in mM: 124.0 NaCl, 4.4 KCl, 26.0 NaHCO_3_, 1.0 NaH_2_PO_4_, 2.5 CaCl_2_, 1.3 MgSO_4_, 10 glucose)^44^. After slicing, the chamber was removed from the water bath, and slices were permitted to recover from the mechanical shock of slicing for at least 90 min at room temperature before recording.

### Experimental design

In Experiment 1, we sought to determine the extent to which HFD disrupts late-phase LTP in the hippocampus of young adult and aged rats. For this, rats were assigned to the following conditions: young chow (slices = 9, *n* = 6), young HFD (slices = 6, *n* = 5), aged chow (slices = 10, *n* = 6), and aged HFD (slices = 6, *n* = 4). After brains were collected and processed as described above, we proceeded to perform extracellular field recordings for late-phase LTP (L-LTP). Field excitatory postsynaptic potentials (fEPSPs) were recorded from Schaffer collateral–CA1 synapses by placing both stimulating and recording electrodes in the stratum radiatum. All stimuli were delivered at intensities that evoked fEPSP peak amplitude ~35–50% of the maximum response recorded during I/O measurements in each slice. The percentage of facilitation of PPF was calculated from the ratio of the second fEPSP peak amplitude to the first fEPSP peak amplitude, shown at intervals ranging from 50 to 250 ms. Test stimuli were delivered once every minute, and test responses were recorded for 15–30 min before beginning the experiment to assure stability of the response. The same stimulus intensity was used for LTP induction and to evoke test responses. Theta-burst protocol (12 bursts of 4 pulses at 100 Hz, delivered 200 ms apart) was used to induce L-LTP, and the recording lasted two hours. fEPSP mean peak amplitude was used as a measure of synaptic activity.

In Experiment 2, we determined the extent to which HFD disrupts early-phase LTP in aged rats. In a separate cohort of animals from Experiment 1, rats were assigned to the following conditions: aged chow (slices = 8, *n* = 6) and aged HFD (slices = 8, *n* = 6). After tissue collection and slice preparation, fEPSPs were recorded following the same guidelines for location and test stimuli described in Experiment 1. E-LTP was induced by a relatively weak stimulus protocol (a single, 1 s, 100 Hz stimulus train)^43^, and recording lasted one hour.

Lastly, in Experiment 3, we determined the extent to which central IL-1β mediates HFD-induced LTP disruptions in aged rats. Rats were assigned to the following conditions: chow saline (slices = 9, *n* = 5), chow IL-1RA (slices = 7, *n* = 6), HFD saline (slices = 7, *n* = 5) and HFD IL-1RA (slices = 8, *n* = 6). IL-1RA (Kineret) was injected at a dose of 112 μg/3 μL/rat to block IL-1 signaling known to be evoked by HFD, as we have previously done^24^. IL-1RA or an equal volume of sterile saline was administered via intracisterna magna injection, a minimally invasive procedure that does not require surgery or cannula placement, which could provoke memory impairments in aging animals^50,70^. Also, when injected centrally, IL-1RA has a half-life of 4 days, significantly longer than its 90-min half-life when injected peripherally^51,52^. On day 1 (prior to receiving their assigned diet), rats were anesthetized with isoflurane, and the dorsal posterior aspect of the head was shaved and swabbed with 70% EtOH. A 27-gauge needle, connected to a 25-μl Hamilton syringe via PE50 tubing, was inserted into the cisterna magna. Following their injection of either IL-1RA or saline, rats were returned to their home cage where they received their assigned diet. After tissue collection and slice preparation, as described above, LTP procedures as described in Experiment 1 were performed.

### Data compilation and statistical analysis

All data are presented as means + SEM. Electrophysiology data acquisition and analysis software were performed with pCLAMP 10 and 11 software. The percentage of facilitation of PPF was calculated from the ratio of the second fEPSP peak amplitude to the first fEPSP peak amplitude. LTP under TBS was normalized to the mean peak amplitude fEPSP of 20 min baseline. We recorded EPSP every minute, then we averaged every 5 min across time and these means were used for statistical analyses. Statistical analyses were computed using GraphPad Prism version 9 and StatView. Two-way repeated measures ANOVAs were run for all experiments. In the case of significant interactions, Scheffe’s post hoc tests were run. The threshold for significance was set as *P* < 0.05.

### Reporting summary

Further information on research design is available in the Nature Research Reporting Summary linked to this article.

## Supplementary information

Reporting Summary

## Data Availability

All data supporting the findings of this study are available within the paper.
